# Significance of long chain polyunsaturated fatty acids in human health

**DOI:** 10.1186/s40169-017-0153-6

**Published:** 2017-07-27

**Authors:** Rafael Zárate, Nabil el Jaber-Vazdekis, Noemi Tejera, José A. Pérez, Covadonga Rodríguez

**Affiliations:** 1 Canary Islands Cancer Research Institute (ICIC), Ave. La Trinidad 61, Torre A. Arévalo, 7th floor, 38204 La Laguna, Tenerife Spain; 2 Centre Algatech, Institute of Microbiology, Academy of Sciences of the Czech Republic, Třeboň, Czech Republic; 3 Department of Nutrition and Preventive Medicine, Norwich Medical School, University of East Anglia, Norwich, NR4 7UQ UK; 4 Department of Animal Biology, Soil Science and Geology (Animal Physiology Unit), Faculty of Sciences, Universidad de La Laguna, Ave. Astrofísico Francisco Sánchez s/n, 38206 La Laguna, Tenerife Spain; 5 Institute of Biomedical Technologies (ITB), Universidad de La Laguna, Campus de Ofra, 38071 La Laguna, Tenerife Spain

**Keywords:** Disease, Health, Inflammation, Lipidomics, Lipids, Long chain polyunsaturated fatty acids, Omega-3, Resolvins

## Abstract

In the last decades, the development of new technologies applied to lipidomics has revitalized the analysis of lipid profile alterations and the understanding of the underlying molecular mechanisms of lipid metabolism, together with their involvement in the occurrence of human disease. Of particular interest is the study of omega-3 and omega-6 long chain polyunsaturated fatty acids (LC-PUFAs), notably EPA (eicosapentaenoic acid, 20:5n-3), DHA (docosahexaenoic acid, 22:6n-3), and ARA (arachidonic acid, 20:4n-6), and their transformation into bioactive lipid mediators. In this sense, new families of PUFA-derived lipid mediators, including resolvins derived from EPA and DHA, and protectins and maresins derived from DHA, are being increasingly investigated because of their active role in the “return to homeostasis” process and resolution of inflammation. Recent findings reviewed in the present study highlight that the omega-6 fatty acid ARA appears increased, and omega-3 EPA and DHA decreased in most cancer tissues compared to normal ones, and that increments in omega-3 LC-PUFAs consumption and an omega-6/omega-3 ratio of 2–4:1, are associated with a reduced risk of breast, prostate, colon and renal cancers. Along with their lipid-lowering properties, omega-3 LC-PUFAs also exert cardioprotective functions, such as reducing platelet aggregation and inflammation, and controlling the presence of DHA in our body, especially in our liver and brain, which is crucial for optimal brain functionality. Considering that DHA is the principal omega-3 FA in cortical gray matter, the importance of DHA intake and its derived lipid mediators have been recently reported in patients with major depressive and bipolar disorders, Alzheimer disease, Parkinson’s disease, and amyotrophic lateral sclerosis. The present study reviews the relationships between major diseases occurring today in the Western world and LC-PUFAs. More specifically this review focuses on the dietary omega-3 LC-PUFAs and the omega-6/omega-3 balance, in a wide range of inflammation disorders, including autoimmune diseases. This review suggests that the current recommendations of consumption and/or supplementation of omega-3 FAs are specific to particular groups of age and physiological status, and still need more fine tuning for overall human health and well being.

## Introduction

The term ‘omics’ and the technologies associated with have expanded rapidly with the completion of the human genome in the early 2000s. “Genomics”, which refers to the multidisciplinary technology developed to determine the structure, function, evolution, and mapping of genomes was the initial “omic” [1]. This was followed by many others, such as transcriptomics, proteomics and peptidomics, metabolomics, and lipidomics [2, 3]. The latter is a subset of the metabolomics, and aims at mapping, determining and quantifying all lipids within healthy or altered cells, tissues or organisms [4, 5].

Lipidomics emerged about 15 years ago, and its progress has depended directly on the advances made in analytical technologies, particularly gas chromatography (GC) and liquid chromatography (LC). The ability of both techniques to be coupled with mass spectrometry (MS) and the different ionization technologies developed including electrospray ionization (ESI), matrix-assisted laser desorption/ionization (MALDI), and atmospheric pressure chemical ionization (APCI) has vastly improved their sensitivity. Other techniques, such as nuclear magnetic resonance (NMR), have also been utilized. All are accompanied by robust software to process the vast amounts of data collected [6, 7]. Thus, the field of lipid research is clearly advancing in parallel with the progress of analytical techniques. These advances allow the determination of lipid profile alterations and how these are associated with diseases. They can also detect changes in lipid metabolism or pathway modulation by lipids, that can now be established even in complex biological systems. This provides new insights, enabling the discovery and characterization of molecular biomarkers, as well as the understanding of various pathologies associated to lipids and their mechanisms, for the subsequent restoration of lipids, prevention, drugs design and treatment of these ailments by acting on the lipid impact or alteration.

Lipids are natural products derived from polyketides, which in turn originate from the acetate route [8]. The term lipids, includes fatty acids (FAs) and their derivatives, as well as related functional or biosynthetic substances derived from these compounds. Lipids are indispensable elements in the diet, both for the provision of energy (beta-oxidation of FAs), and for the supply of essential FAs, i.e. ALA (alpha-linolenic acid, 18:3n-3) and LA (linoleic acid, 18:2n-6) [9]. They have biological activities that act to influence the function and responsiveness of cell membranes and tissue metabolism, to hormonal and other signals. These biological activities may be grouped as regulation of membrane structure and function, regulation of intracellular signalling pathways, transcription factor activity together with gene expression, and regulation of the production of bioactive lipid mediators. Through these actions, FAs affect health, well-being, and the risk of developing disease [10].

FAs are the simplest lipids, which in turn are components of other more complex lipids. FAs are organic compounds containing a hydrophilic carboxyl group attached to a hydrocarbon chain varying from C6 (six carbon atoms) to C32, with an additional terminal methyl group. Most of the FAs existing in nature have an even number of carbon atoms and linear hydrocarbon chains, although some of them, found primarily in bacteria, may contain branching or even cyclic structures [8]. In addition, these chains may be presented in two main forms, saturated without the presence of double bonds, or unsaturated, containing one or more double bonds, the latter being of greater physiological importance because of their medicinal properties. Of particular interest within the unsaturated family are the long chain polyunsaturated FAs (LC-PUFAs), which can be categorized into two principal families—omega-3 (n-3) and omega-6 (n-6)—depending on the position of the first double bond from the methyl end group of the FA. Common LC-PUFAs are EPA (eicosapentaenoic acid, 20:5n-3), DHA (docosahexaenoic acid, 22:6n-3) and ARA (arachidonic acid, 20:4n-6).

**Fig. 1:**
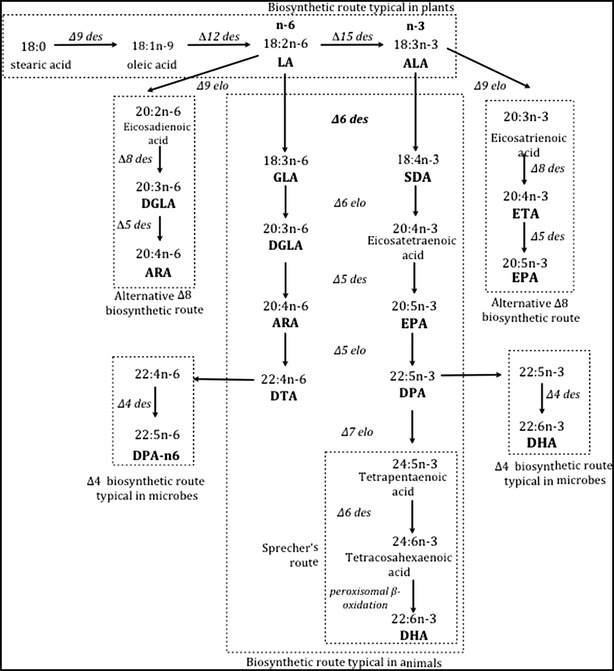
Biosynthetic route of fatty acids requiring the essential LA (linoleic acid) and ALA (alpha-linolenic acid) with the different variations and steps. *des* desaturase, *elo* elongase; ARA (arachidonic acid), DGLA (dihomo-gamma linolenic acid), DHA (docosahexaenoic acid), DPA (docosapentaenoic acid), DTA (docosatetraenoic acid), EPA (eicosapentaenoic acid), ETA (eicosatetraenoic acid), GLA (gamma-linolenic acid), SDA (stearidonic acid)

The synthesis of FAs can proceed either by anaerobic routes through polyketide synthase enzymes, which are only present in some microorganisms, or by aerobic metabolic pathways, which are more universal, as they occur in plants, algae, fungi, and animals. This consists of successive elongation (carbon chain elongation) and desaturation steps (inclusion of double bonds into the carbon chain), reactions controlled by elongase and desaturase enzymes [11] (Fig. 1).

There are variations in the order in which the steps of desaturation and elongation occur. Thus, “metabolic pathway 6” begins with a ∆6-desaturation, followed by chain elongation and desaturation thereof, to yield EPA when the initial substrate is ALA, or to yield ARA when the initial substrate is LA. Next, EPA is elongated to DPA (docosapentaenoic acid, 22:5n-3), which is desaturated again in the 4-position to finally produce DHA (Fig. 1). There also exists the so-called Sprecher’s route, which describes the production of DHA by consecutive Δ6 and Δ5 desaturations of ALA, to produce C24 metabolic intermediates that are finally shortened to DHA by a β-oxidation step that takes place in the peroxisomes [12]. What defines this route, which has been characterized in mammals and fish [13, 14], is that the final product, DHA, is synthesized without the intervention of the Δ4-desaturase. Finally, the “alternative route 8”, typical in protists and algae, begins directly with an elongation step from C18 to C20, followed by desaturations at positions 8 and 5, generating DGLA (dihomo-γ-linolenic acid, 20:3n-6) and ARA, respectively, as well as to finally produce ETA (eicosatetraenoic acid, 20:4n-3) and EPA [15] (Fig. 1).

Diet, among other factors, has been postulated to modulate human capacity to produce LC-PUFAs from their essential C18 FA precursors, i.e. ALA and LA [16]. However, most recent studies indicate that endogenous synthesis of DHA from ALA in humans is much lower and more limited than previously assumed [17, 18] and as a consequence, sources of n-3 LC-PUFAs such as fish should be included in the human diet for general health [19, 20].

Thereby, with the technical advances attained in the last decades, and the deeper understanding of cell membrane physiology, lipids have eventually gained importance in maintaining health, and more importantly, in their involvement in the development of diseases. In this review, we demonstrate the use of lipidomics and the corresponding analytical tools to highlight the most recent information and evidences that LC-PUFAs participate in several major human pathologies.

## Inflammatory-autoimmune diseases and lipids

Virtually all diseases including diabetes and obesity, cancer and cardiovascular, and neurodegenerative diseases, manifest some symptoms of inflammation. Other disorders classified under the term of inflammatory diseases, such as allergies, asthma, arthritis and autoimmune diseases, are dramatically increasing in occurrence. Possible causes of these inflammatory conditions are pointing to some novel factors, such as hidden allergens and infections, environmental and dietary toxins, intake of pro-inflammatory diets, and even sustained stress.

Western countries seem to be facing epidemics of allergic (60 million people), asthmatic (30 million people) and autoimmune disorders (24 million people), which include a wide variety of syndromes affecting all together around 5 percent of the total Western population [21, 22]. Rheumatoid arthritis, lupus, multiple sclerosis, psoriasis, celiac disease, inflammatory bowel disease, ulcerative colitis, Crohn’s disease and thyroid disease, are just some of the ailments that belong to this poorly understood group of diseases. They are twenty first century illnesses that mostly occur in developed countries.

Autoimmune disorders are characterized by a systemic inflammation, which results in the body attacking its own tissues. In the absence of an infection or other clear reason, the activation of T cells or B cells or both, gives rise to this clinical condition. It seems that women are more susceptible to suffer from autoimmune diseases, and that environmental factors and alien substances may act as haptens and render autoantigens immunogenic. Autoimmune syndromes and their multifarious and still unknown foundations are a challenge to the progress and testing of new therapies to fight disease [22, 23].

Although an attempt to decrease total fat and saturated fat intake in Western diets has become a dietary tonic, with evolution, the intake of omega-6 FAs has increased while that of omega-3 FAs has decreased. As a result, the omega-6/omega-3 ratio has substantially risen from 1:1 in our ancestral diets, to around 20:1 in today’s Western diets. This change also coincides with a significant increase in the prevalence of overweight and obesity, together with a wide range of inflammation disorders. However, controversial results have been reported regarding the influence of the dietary ratio of omega-6/omega-3 FAs on body fat increments, mechanisms of adipogenesis, browning of adipose tissue, lipid homeostasis, brain–gut-adipose tissue axis, and systemic inflammation [24, 25].

The reduction of inflammation observed in clinical studies with supplemental omega-3 LC-PUFAs opens new options to pharmacologically treat systemic lupus erythematosus [26]. Additionally, animal models demonstrate the benefits of n-3 FAs in rheumatoid arthritis (RA), inflammatory bowel disease (IBD) and asthma. Whereas results using supplemental fish oil in patients with IBD are still unreliable, clinical trials involving patients with RA have demonstrated benefits that have been further supported by meta-analyses studies [27].

Globally, experimental results highlight that a wide number of inflammation-related events can be partly impeded by a dietary intake of EPA and DHA from oily fish and fish oil supplements. As Calder elegantly revised [28, 29], these events include leukocyte chemotaxis, cellular adhesion molecule (CAM) expression, leukocyte–endothelial adhesive interactions, production of ARA derived eicosanoids like PGs and LTs, production of inflammatory cytokines and T cell reactivity. Mechanisms underlying the anti-inflammatory actions of n-3 LC-PUFAs also include: alteration of cell membrane phospholipid FA composition, disruption of lipid rafts, inhibition of the activation of the pro-inflammatory nuclear transcription factor kappa B (NF-κB), activation of the anti-inflammatory transcription factor NR1C3 (i.e. peroxisome proliferator activated receptor), and binding to the G protein coupled receptor GPR120. Mechanisms which are all interlinked.

**Fig. 2:**
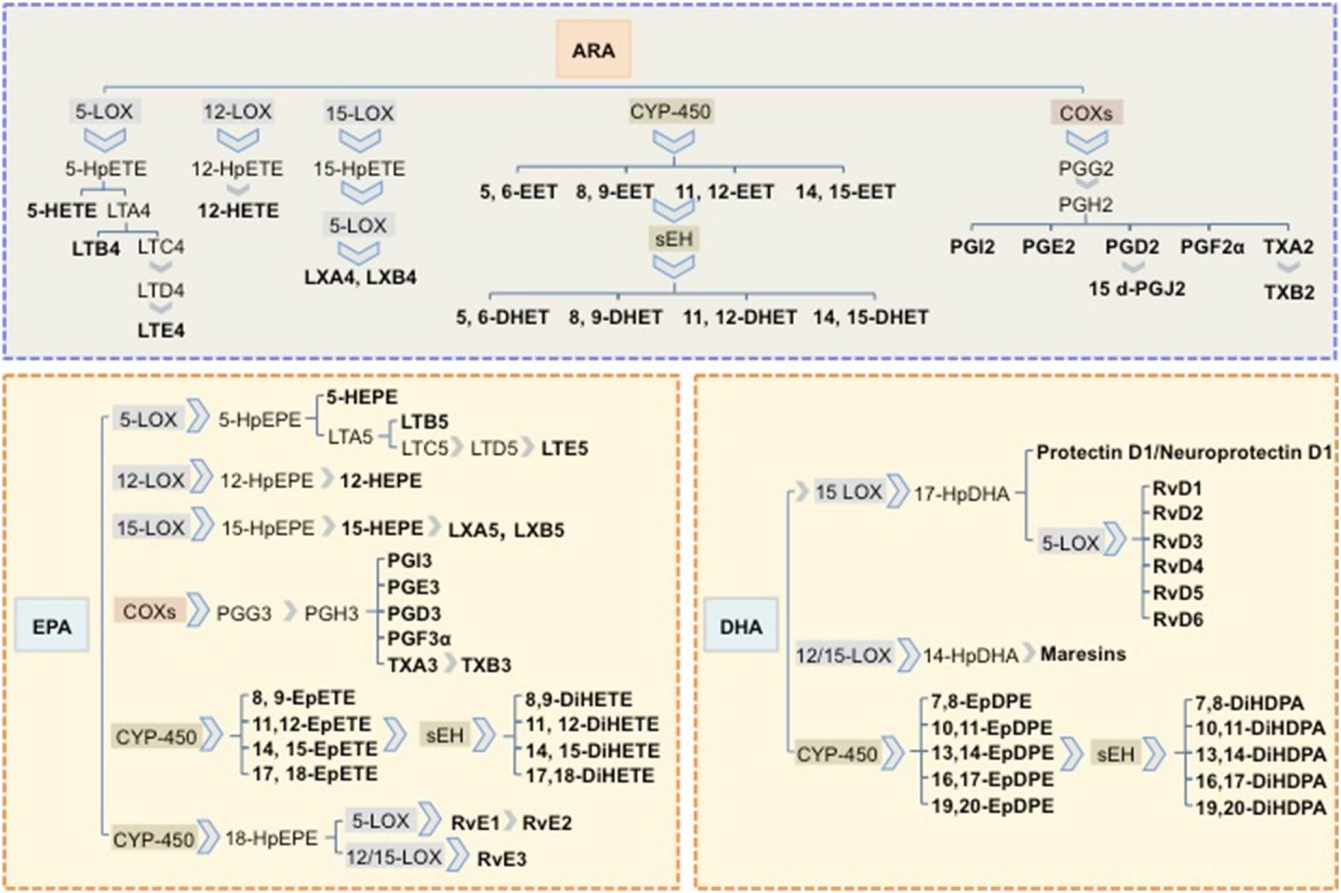
Principal bioactive lipid mediators derived from ARA, EPA and DHA by the action of lipoxygenases (LOXs), cyclooxygenases (COXs), cytochrome P450 (CYP-450) and soluble epoxide hydrolase (sEH). ARA (arachidonic acid), DHA (docosahexaenoic acid), DHET (dihydroxy-eicosatrienoic acid), DiHDPA (dihydroxy-docosapentaenoic acid), DiHETE (dihydroxy-eicosatetraenoic acid), EET (epoxy-eicosatrienoic acid), EPA (eicosapentaenoic acid), EpDPE (epoxy-docosapentaenoic acid), EpETE (epoxy-eicosatetraenoic acid), HEPE (hydroxy-eicosapentaenoic acid), HETE (hydroxy-eicosatetraenoic acid), HpDHA (hydroperoxy-docosahexaenoic acid), HpEPE (hydroperoxy-eicosapentaenoic acid), HpETE (hydroperoxy-eicosatetraenoic acid), LT (leukotriene), LX (lipoxin), PG (prostaglandin), Rv (resolvins), TX (thromboxane)

PUFAs can regulate a wide set of homeostatic and inflammatory processes linked to numerous diseases either directly or via transformation into locally acting bioactive metabolites [30]. These metabolites are products of reactions with cyclooxygenases (COX1 and COX2), lipoxygenases (LOX), cytochrome p450 (CYP-450), or other downstream enzymes, such as soluble epoxide hydrolase (sEH) and non-enzymatic reactions [31–34] (Fig. 2). The physiological functions of these PUFA-derived lipid mediators are only well understood for a few molecules. It seems that some of the agonists and receptors that regulate inflammation, and the cytokine cascade that accompanies infection, initiate the release of ARA and related PUFAs, resulting in a lipid bioactive signalling storm [35–37].

Classically, the term eicosanoids has been used to describe not only those metabolites derived from ARA, but also from other 20 carbon PUFAs, such as EPA and DGLA. Eicosanoids include the COX products prostaglandins (PGs), prostacyclin (PGI) and thromboxanes (TXs); the LOX derived leukotrienes (LTs) and lipoxins (LXs), and the CYP-450 hydroxyeicosatetraenoic acids (HETEs) and epoxyeicosatrienoic acids (EETs) (Fig. 2). Eicosanoids derived from omega‐6 PUFAs (i.e. ARA products PGE_2_ and LTB_4_) are more powerful than their omega-3 PUFAs counterparts (i.e. EPA derived PGE_3_ and LTB_5_), which exert anti-inflammatory effects. SDA (stearidonic acid, 18:4n-3) and its elongated product 20:4n-3, have been reported to produce low active and health promoting eicosanoids able to lower plasma triglycerides (TG) [38].

Not only the absolute amounts of omega-6 and omega-3 FA intake, but also the above mentioned increased omega-6/omega-3 ratio, have been recently shown to be highly pro-thrombotic and pro-inflammatory, and contribute to the prevalence of atherosclerosis, obesity, diabetes and inflammatory-autoimmune diseases [25]. The larger the intake of ARA or its C18 precursor LA, the higher the production of their respective derived pro-inflammatory eicosanoids.

In the last two decades, new families of lipid mediators important in the resolution of inflammation have been discovered and are being investigated. Thus, resolvins, protectins and maresins (Fig. 2) have been identified in the resolving exudates of acute inflammation. EPA-derived E-series (RvE) and DHA-derived or D-series (RvD) resolvins display potent anti-inflammatory and immunoregulatory actions that include blocking the production of pro-inflammatory mediators and regulating trafficking of leukocytes [34]. In addition to the D-series resolvins, DHA is also a precursor of other anti-inflammatory docosanoids named protectins (PDs) and maresins. These pro-resolving lipid mediators, together with the above mentioned resolvins, and the anti-inflammatory lipoxins, have been grouped together as specialized pro-resolving mediators (SPM). These are interesting compounds that constitute a novel topic of research, not only due to their bioactive role in the “return to homeostasis” process, but also elucidating the physiological functions of other n-3 LC-PUFAs. In this sense, SPMs could be responsible for the efficacy of n-3 LC-PUFAs in the prevention and amelioration of childhood asthma, food allergies or atopy [39].

Metabolism of most eicosanoids/docosanoids implies the release of FAs esterified into phospholipids, by phospholipase A2 (PLA2) enzymes. Consequently, increased levels of free FAs and lipid mediator biosynthesis occur particularly after inflammatory cell activation. The most frequently involved PLA2s in the cellular production of bioactive lipids are, the cytosolic calcium-dependent PLA2 (cPLA2), the cytosolic calcium-independent PLA2 (iPLA2), and the secretory PLA2 (sPLA2). Among them, cPLA2, shows a preference to hydrolyse ARA in the *sn*-2 position of phospholipids, and is generally considered the dominant enzyme mediating production of pro-inflammatory eicosanoids, as well as the release of related n-3 PUFAs, i.e. EPA, DPA and DHA bioactive derivatives. In contrast, through the production of lysophospholipids iPLA2 plays a key role in most daily cellular functions, particularly in membrane homeostasis and remodelling. Finally, it remains unclear what role sPLA2 may play in eicosanoids signalling, although a cPLA2-dependent increase in the magnitude and duration of free FA levels, including ARA caused by sPLA2 up-regulation has been recently proposed [37, 40].

As already mentioned, the eicosanoids derived from ARA as a substrate are generally far more potent as pro-inflammatory and pro-thrombotic signalling regulators than those from EPA and DGLA. PGE2 and LTB4 in particular induce the production of interleukins IL-6 and IL-1β, and tumour necrosis factor (TNF). It is also known that the mechanism for PGE2-induced IL-6 production is via direct activation of the nuclear transcription factor *kappa B* (NF-κB). However, at the same time, DHA is a potent regulator of NF-κB by several mechanisms, since it can directly inhibit NF-κB activation, or prevent its activation by up-regulating intracellular glutathione to levels that effectively balance the oxidative stress. Furthermore, indirect mechanisms for DHA include its oxidation to the above mentioned potent signalling molecules, the resolvins. During conditions of tissue stress, both EPA and DHA may be released from phospholipids by PLA2 to undergo conversions to resolvins that actively promote the resolution of inflammatory processes. Known anti-inflammatory mechanisms for the resolvins include the down-regulation of NF-κB, and the removal of neutrophils from inflammatory sites [19].

Since the introduction of lipidomics, the expanded view of pro-inflammatory and anti-inflammatory contributions of n-3 PUFAs derived eicosanoid/docosanoid signalling is remarkably more complex than initially thought. However, the potential to understand and develop novel treatments for proper inflammatory and metabolic conditions have become extremely promising. The beneficial properties of omega-3 FAs have been appreciated for many years. However, the use of new lipidomics analytical strategies, with increased sensitivity and throughput, has identified new FA-derived bioactive metabolites with new subjacent molecular mechanisms of action. Undoubtedly, this area of research has further highlighted the importance of eicosanoids in the inflammatory process, and in particular, the role of omega-3 metabolites in controlling the inflammatory response and switching inflammation into a resolution state. In addition, it has also revealed the importance of gaining a ‘global’ view of eicosanoid and docosanoid metabolism in order to better understand the complex and exquisitely regulated mechanisms of their generation, action and metabolic inactivation, as well as an understanding into how these interconnecting and tightly regulated metabolic pathways are deregulated in disease settings [41].

A better comprehension of the cytokine cascade and its integration with the eicosanoid and docosanoid eruption that accompanies inflammation and its resolution should provide new insights leading to novel strategies for the understanding and treatment of inflammation according to individual age, genetic variation or dietary regime [37]. Meanwhile, the tremendous importance of acquiring the beneficial omega-3 PUFAs through diet to mitigate or prevent the appearance of these ailments has also been presented.

## Lipids and cardiometabolic health

Cardiovascular disease (CVD) was the leading global cause of death in 2013, claiming more lives each year than all forms of cancer and chronic lower respiratory disease combined. According to the last American Heart Association Heart Disease and Stroke Statistics Report, CVD accounted for over 17.3 million deaths per year globally, a number that is expected to grow above 23.6 million by 2030 [42].

**Fig. 3:**
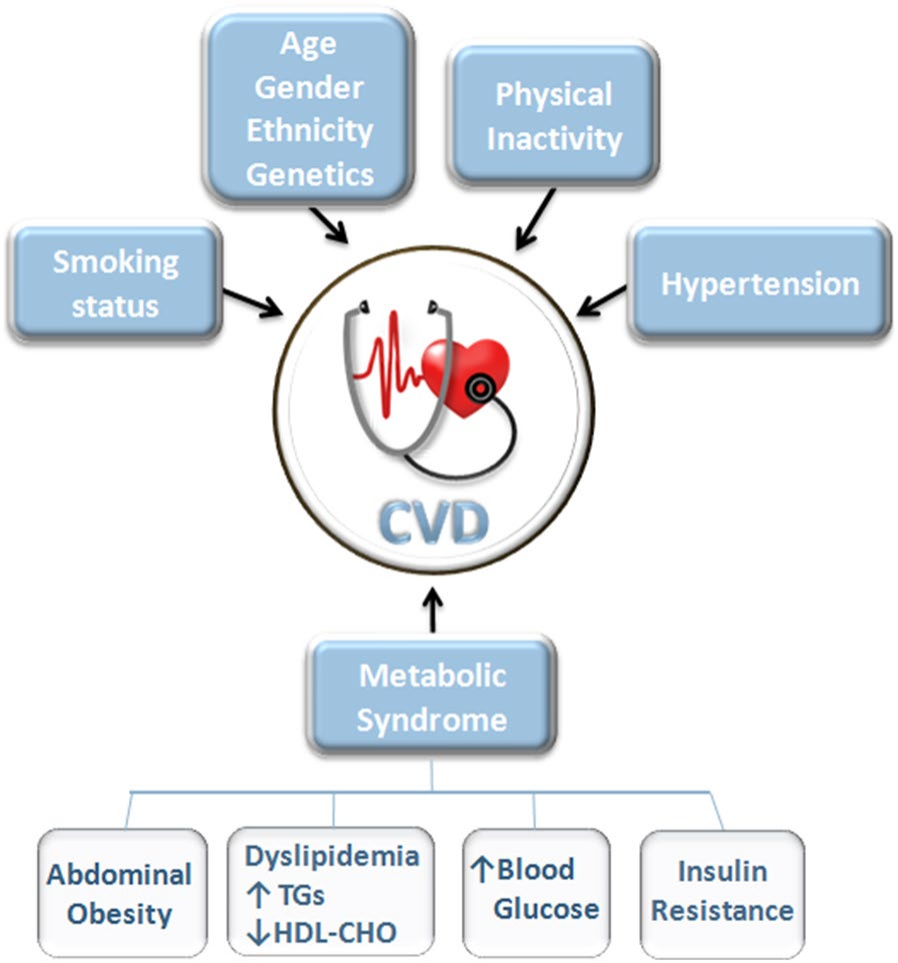
Scheme of cardio-metabolic risk factors associated with the prevalence of cardiovascular disease (CVD)

Several cardio-metabolic risk factors are associated with the prevalence of CVD, with only few of them, such as age, gender, genetics or family history, being non-modifiable. Most well-established risk factors for CVD are behavioural or modifiable, and are the target of preventive or treatment interventions [43]. These risk factors include tobacco smoking, physical inactivity, inadequate diet, high blood pressure (SBP/DBP ≥140/90 mm Hg) and those that fall under the umbrella of metabolic syndrome, i.e. high fasting plasma glucose (≥100 mg/dL), dyslipidemia (triglycerides (TG) ≥150 mg/dL and HDL-C <40 mg/dL in males or <50 mg/dL in females), obesity (waist circumference >102 cm in males or >88 cm in females) and insulin resistance [42] (Fig. 3).

Within the possible lifestyle modifications for the primary and secondary prevention of CVD, following a cardioprotective diet constitutes one of the main recommendations of many health and nutritional organizations and governing bodies. Consumption of fruits, vegetables, dietary fibre, and monounsaturated and polyunsaturated fats coming from sources such as fish, nuts, and vegetable oils, has been shown to have a protective effect [44].

Multiple observational studies and early-randomized clinical trials show an inverse relationship between the ingestion of marine derived omega 3 or n-3 LC-PUFAs and major cardiovascular adverse outcomes [45]. Recommendations of n-3 LC-PUFAs intake from organizations such as the International Society for the Study of Fatty Acids and Lipids (ISSFAL), the United Kingdom Scientific Advisory Committee in Nutrition (SACN) and the American Heart Association (AHA) include the consumption of 2–3 portions of oily fish per week in order to provide a minimum dietary intake of 200–500 mg/day of EPA plus DHA, increasing to 1–4 g/day for the secondary prevention of CVD or as a TG lowering therapy [46–48].

The capacity of n-3 LC-PUFAs to lower fasting plasma TG concentrations and VLDL production, one of the main TG-transporting lipoproteins, is one of the main proposed mechanisms that would explain omega-3 LC-PUFAs cardioprotective role, and it is also one of the most consistent findings across studies. Taking EPA and/or DHA in doses of 3.4–4 g/day have been shown to decrease TG levels by an average of 29% in individuals with levels higher than 150 mg/dL [49].

Proposed mechanisms to account for these effects include changes in transcription of several nuclear receptors involved in controlling lipid homeostasis: sterol regulatory element binding proteins (SREBP), liver X receptor-alpha (LXRα), retinoid X receptor alpha (RXRα), farnesoid X receptor (FXR), and peroxisome proliferator-activated receptors (PPARs). EPA and DHA have been shown to regulate SREBP1c expression by inhibiting binding of the LXR/RXR heterodimer to the LXREs in the SREBP-1c promoter. This inhibition is translated into a decrease in de novo lipogenesis and therefore, into a reduction of circulating TG levels [29, 49].

PUFAs also inhibit transcription of HNF-4α, regulating fat and carbohydrate metabolism and bile acid synthesis. Binding of n-3 LC-PUFAs activate FXR, which reduces TG levels by increasing hepatic clearance through modulating lipoprotein lipase activity, inducing PPARα, and inhibiting SREBP-1c. Importantly, activation of PPARα increases FA oxidation, lipoprotein lipase, FA transport and enhances adipocyte differentiation, all contributing to lowering TG [45].

Along with their lipid-lowering properties, omega-3 LC-PUFAs exert other cardioprotective functions, i.e. reduction of platelet aggregation and inflammation, antiarrhythmic effect, improvement of endothelial function and myocardial relaxation and efficiency, stabilization of atherosclerotic plaques, and reduction of resting heart rate and systolic and diastolic blood pressure [50]. The beneficial effects of EPA and DHA in reducing hypertension have been reported in both animal and human studies. Several meta-analyses of placebo controlled trials show reductions in blood pressure in hypertensive, as well as in normotensive individuals [51].

With reference to other cardiovascular risks also associated with CVD and metabolic syndrome, protection against the development of impaired glucose homeostasis and insulin resistance has been reported in animal studies, especially in murine models of metabolic syndrome and type-2 diabetes. n-3 LC-PUFAs increase the expression of insulin-stimulated glucose transporter-4 (GLUT4), insulin receptor substrate-1 (IRS1) and glycogen synthase-1 (GYS1) in skeletal muscle, enhancing glucose utilization [52]. In humans however, it is still not clear whether n-3 LC-PUFAs have clinically relevant effects on insulin resistance or diabetes risk.

Regarding lipid mediators and CVD, as described previously, eicosanoids have been attributed to a diverse range of biological activities, with PGs, TBXs and LTs displaying pro-inflammatory or pre-thrombotic roles. PGE_2_, in addition of being involved in all processes leading to the classic signs of inflammation (redness, swelling, and pain); it is also known to play a key role in the pathogenesis of cardiovascular diseases, contributing to regulation of blood pressure. TXA_2_ has been shown to promote platelet aggregation, vasoconstriction, and smooth muscle proliferation, while leukotriene LTB4 promotes atherosclerosis [53]. However, not all ARA metabolites have a counterproductive effect, with some of them being able to display a beneficial/detrimental role depending on the tissue they are targeting or just having a general positive effect. In particular, ARA metabolites like EETs and their corresponding diols (DHETs), LXs and PGI2 have been understood to play a protective role in cardiovascular health (Fig. 2). Thus, EETs decrease inflammation and platelet aggregation, and in general act to maintain vascular homeostasis, while DHETs have vascular activity in coronary arteries. LXs have a number of direct actions on endothelial cells that are protective, and are in line with their role in resolution. PGI2 potently inhibits platelet aggregation and displays powerful vasodilator effects via relaxation of smooth muscle [50, 54, 55].

In addition to this “classic” pathway, EPA and DHA are likely to have a further protective function in cardiovascular health due to their ability to generate specialized pro-resolving mediators (SPMs) named resolvins, protectins and maresins, which display an active role in the resolution of inflammatory processes, [56]. EPA and DHA also serve as substrates for CYP-450 and sEH enzymes, generating epoxides and diols similar in structure but more potent than their ARA analogues (Fig. 2). It appears that DHA-epoxides are particularly efficient in reducing angiotensin-II driven blood pressure [57], and can be further metabolized to their corresponding diols, which are also vasomodulators and display direct effects on vascular smooth muscle tone [58].

Multiple epidemiological studies, prospective cohort studies and randomized controlled trials have provided evidence for the cardioprotective role of n-3 PUFAs. However, the overwhelming beneficial outcome of early studies appears to conflict with the results of recent prospective trials and meta-analysis of clinical trials, where mixed or less favourable results have been reported [59–61].

Back in 1970 epidemiological studies conducted in Greenland Inuits suggested that the high content of n-3 LC-PUFAs in their diet could be associated with the decreased risk of cardiovascular events and cardiovascular mortality observed in this population [62]. Following these observations, several prospective studies were designed to study the effect of fish or fish oil consumption on CVD. The DART study (Diet and Reinfarction Trial) enrolled male myocardial infarction (MI) survivors who were given dietary advice to eat at least two servings of fatty fish/week or fish oil capsules (0.5 g/day) for 2 years [63]. The results from this study showed that fish oil consumption reduced mortality by 29%. Another major study, the GISSI (Gruppo Italiano per lo Studio della Sopravvivenza nell’ Infarto Miocardico-Prevenzione Trial) [64], showed an impressive reduction in both total and CVD mortality in post-MI patients taking 850 mg/day of EPA/DHA in a 1:2 ratio. However, a follow up of this trial, the GISSI-Heart Failure study [65], investigated the effect of 1 g/day omega-3 PUFA in patients with heart failure, reported a smaller reduction in mortality compared with the GISSI-P outcome.

The JELIS study (Japanese Eicosapentaenoic Acid Lipid Intervention Study), an open-label blinded trial conducted with patients with hypercholesterolemia, showed that 1.8 g/day of EPA reduced combined cardiovascular events, although no effect on the incidence of arrhythmia was observed [66].

Recently, the double-blind Alpha-Omega Trial [67] indicated that an additional low-dose omega-3 supplementation did not significantly reduce the rate of major cardiovascular events in post-MI patients who were receiving state-of-the-art antihypertensive, antithrombotic, and lipid-modifying therapy. However, a secondary analysis in those patients with diabetes suggested that those who received the EPA-DHA plus ALA treatment experienced less ventricular arrhythmia-related events, and less combined end-point ventricular arrhythmia-related events. They also had a reduced number of fatal MIs [68].

In the last 7 years, other studies including the OMEGA trial, the SU.FO.OM3 trial, the ORIGIN study, the Risk and Prevention Study, and the AREDS2 trial have provided little or no evidence that n-3 PUFAs consumption reduces cardiovascular events, mortality or morbidity [69].

An explanation of the discrepancy between the results obtained in early trials and more recent studies could be the differences in baseline levels of omega-3 and drug therapy status of participants. Most of the early studies were carried out in the pre-statins era and when consumption of fish oil was not as widespread, so it is understood that higher levels of n-3 PUFAs in the current background diet and higher numbers of participants on CVD medications would make it more difficult for n-3 PUFAs to display a significant effect on CVD outcomes.

In this regard, the erythrocyte membrane EPA and DHA content or omega-3 index has been proposed as an indicator of n-3 LC-PUFAs intake, and as a risk stratification tool for coronary heart disease death. Setting an Omega-3 Index cut off or baseline level during the recruitment of participants would possibly allow outcomes of prospective trials to be more pronounced [70].

With reference to the interaction of drugs used in secondary prevention of CVD and n-3 PUFAs, two clinical trials are being conducted at present, the reduction of cardiovascular events with EPA-Intervention Trial (REDUCE-IT) and STRENGTH (Statin Residual Risk Reduction with Epanova in High Risk Patients with Hypertriglyceridemia) to provide further evidence of the effect of omega-3 supplementation and statin therapy in established CVD or high risk for CVD participants [69].

In addition to the assessment of the n-3 LC-PUFAs baseline levels and the clinical history/medication of the prospective participants, other aspects, such as the quality and stability of the n-3 LC-PUFAs sources, the bioavailability achieved with the selected EPA and DHA chemical form, the selection of the most appropriate FA dosage to use, and the ideal EPA:DHA ratio when these FAs are used in combination, should be taken into account. Other factors as the improved digestion and absorption of these FAs when they are consumed along fatty meals, together with the selection of the most adequate study length and the use of compliance indicators during the course of the intervention, should carefully be weighed as important study design components that could possibly affect the overall outcome of future intervention trials.

## Cancer and lipids

Cancer cells are known to exhibit a high and uncontrollable capacity to divide and proliferate. They require cellular building blocks, having an avidity for certain types of nutrients, particularly sugars, glutamine, nucleic acids, proteins and lipids, the latter being needed for the formation of new membranes [71–73]. The high potential to boost angiogenesis or vascularization mechanisms for the progression of cancer is also well documented [74], as well as their metastatic faculty to invade healthy organs, with metastasis being the cause of 90% of deaths from solid tumours [75].

Regarding lipids, despite being ignored for a long time, an increasing number of reports are being made available recognising the importance of these molecules within the cancer process where lipids, and in particular FAs, are required primarily for building new cell membranes. Cell membranes are composed of complex lipids, many of which are derived from palmitic acid (16:0). Lipids also act as biomolecules with ample signalling and transcription capabilities. Interestingly there are marked differences in the lipid profiles of cancer cells compared with normal cells [76].

Like glutamine and lipids it has been agreed upon that cancer cells exhibit a high uptake of sugars, i.e. glucose, as an energy and building block molecule [71, 72, 77]. This sugar demand has served as a diagnostic tool in fluorodeoxyglucose positron emission tomography (FDG-PET) that can detect and image high glucose metabolism spots within the body [78].

Total sugar consumption positively correlated with the risk of esophageal adenocarcinoma; while added fructose was linked with an increased risk of small intestine cancer. In general, all investigated sugars were related to an increased risk of pleural cancer. Nonetheless, no relation between dietary sugars and risk of colorectal or any other major cancer was found [72]. A classical assumption has been that most proliferating cells rely on de novo synthesis of lipids from sugars to make new cell walls. However, an elegant report exhibits the nutrient preferences of proliferating fibroblasts and cancer cells in the presence of glucose and/or lipids (palmitate). Standard growth media for animal cells contain relatively low level of lipids, but large amounts of sugars and proteins. Employing this type of growth medium, fibroblast cells, HeLa and H460 cancer cells rely primarily in de novo synthesis of lipids from glucose or glutamate. However, when exogenous palmitate was added to the culture medium, de novo synthesis was very minor, showing a clear preference and marked uptake of palmitate (ca. 90%) from the medium for membrane formation. This evidence supports the importance of exogenous FAs to cellular proliferation. Moreover, blocking FA uptake decreased the proliferation of fibroblast, HeLa and H460 cells, and supplementing them with exogenous palmitate, decreased lipogenic demand and dropped glucose uptake. These results suggest that proliferating cells utilizing exogenous lipids would be less susceptible to drugs designed to inhibit glycolysis and/or lipogenesis for cancer treatment [73].

Cancer cells alter their metabolism to gain a continuous and potent proliferation capability. It has been cited that one major player is the modification of lipid metabolism, together with the reprogramming of lipid synthesis with the activation of many pathways and protein–protein interactions [79, 80]. Lipogenic enzymes, such as acetyl CoA carboxylase (ACC), fatty acid synthase (FASN), ATC citrate lyase (ACLY), are increased in most tumours [77, 81, 82]. FASN appears overexpressed in many cancer types including breast and prostate, suggesting that FA synthesis plays a crucial role in cancer development [79]. Similarly, FA β-oxidation enzymes, particularly carnitine palmitoyltransferase 1 isoforms, were also overexpressed in many human tumours [80], with the upregulation of the FA biosynthesis beginning at early stages in the cancer process in various types of cancers [77].

Analogously, some downstream cancer cell lipid metabolism transcription factors and signal regulating genes are overexpressed; in particular, FASN is controlled by growth factor receptor-associated signalling pathways, including the PI3K-Akt (phosphatidylinositol 3-kinase) and MAPK (mitogen-activated protein kinase), through the SREBP-1 transcription factor [77, 83]. SREBP proteins are central for maintaining cellular lipid homeostasis, and unusual activation of SREBPs can contribute to obesity, fatty liver disease, insulin resistance, and could also be involved in cancer development [83].

Cholesterol is an important component of biological membranes influencing the fluidity of the lipid bilayer. With the reprogramming of cancer cells lipid metabolism, cholesterol synthesis is also affected; for example, in prostate and breast cancers. Its synthesis is highly regulated and proceeds through the mevalonate pathway with 3-hydroxy-3-methylglutaryl-CoA reductase (HMGCR) being a rate-limiting step. HMGCR is the target for statins, which have been reported to show antiproliferative activity in various cancer-cell lines, and have also been shown to increase the sensitivity to chemotherapy agents in colorectal cancer, and patients with acute myeloid leukaemia or hepatocellular carcinoma [79].

In cancer cells major changes in lipid composition, i.e. the presence and/or abundance of saturated vs unsaturated FAs critically affects membrane physiology and plasticity. In bladder cancer tissue, levels of stearic acid (18:0), and oleic acid (18:1n-9) were higher compared to normal tissue, while the level of ARA was lower. Bladder cancer tissue showed a significant reduction in total n-6 PUFAs (−15.1%). The change in the FA composition may be regarded as an indicator of altered lipid metabolism occurring in vivo during human bladder tumorigenesis [84].

In multiple myeloma (MM) patients, significant differences in the plasma FA profile were observed compared to control. The total quantity of saturated and n-6 PUFAs in plasma were significantly higher in the MM group compared to the control group (28.0 vs 25.1%); and more strikingly, total n-3 FAs were significantly decreased in the plasma of patients with MM compared to control (7.7 vs 14.5%). The MM group showed decreased ALA (1.7 vs 6.0%), EPA (2.1 vs 3.8%), and DPA (0.3 vs 1.4%) levels, as well as a clear drop of the n3/n6 ratio (0.3) with respect to the control (0.6) [85]. Compared to normal tissue samples, breast cancer tissue samples had higher levels of monounsaturated FA (oleic acid) and ARA, and lower levels of LA. Furthermore, the breast cancer tissues showed a significantly higher monounsaturated FA/saturated FA ratio [86].

Analogously, adipose tissue also influences cancer progression, particularly for its capacity to act as an endocrine system releasing and controlling biologically active substances (e.g. leptin, adiponectin, TNF-α, etc.) some of which are upregulated in cancer [87]. Moreover, the size and/or number of intracellular lipid stores, or lipid droplets, which mainly contain triacylglycerides and cholesterylesters, are elevated in different types of tumours (breast, prostate, cervical, liver, colon) [79, 88].

Finally, n-3 LC-PUFAs exhibit anticancer activity, whereas n-6 LC-PUFAs promote the development of cancer [89]. Omega-3 LC-PUFAs consumption has been associated with a reduced risk of breast, prostate, colon and renal cancers. On the other hand, the levels of n-6 ARA appeared to be increased in most cancer tissues, while the levels of EPA and DHA were found to be lower than in normal tissues [90]. Rather than individual FAs, the ratio n-6/n-3 is of crucial importance for maintaining a healthy status and for the prevention of cancer, with a 2−4:1 ratio being optimal [91]. The mechanisms involved in the cancer prevention of n-3 LC-PUFAs are thought to proceed via their anti-inflammatory effect, their participation in the TXs pathways, their effects on COX and LOX enzymes, and their effects in many transcription factors [90].

In conclusion, since the role of lipids and/or FAs in cancer has been outlined and has already been demonstrated, it can be inferred that with the progress of lipidomics, understanding the underlying molecular machinery of lipid metabolism, and the role of lipids would assist the discovery of novel and potential targets, and develop new anticancer drugs for cancer therapy [76, 80, 87, 92]. Likewise, knowing the potential of n-3 LC-PUFAs to fight cancer, the inclusion of dietary sources of these PUFAs, such as fish, will direct the balance towards a healthy status rather than a cancer inducing diet characterized by reduced n-3 LC-PUFAs, containing elevated levels of dietary saturated fat, n-6 FAs, and hydrogenated trans-fats as it is often the case.

## Lipids and neurodegenerative disorders

Neurodegeneration is the progressive loss of structure or function of neurons, including death of neurons. Many neurodegenerative diseases including amyotrophic lateral sclerosis, Parkinson’s, Alzheimer’s, and Huntington’s occur as a result of neurodegenerative processes. Aging is a natural process that affects the integrity of the neurovascular system, losing its functions and producing neurodegenerative diseases [93]. Such diseases are incurable, resulting in progressive degeneration and in some instances death of neuron cells. To date, more than 600 neurodegenerative disorders are known. They are classified according to the brain region, affected cell type or the specific molecular markers of each disorder. Genetic studies have discovered a wide range of molecular markers related to neurodegenerative diseases which improves the elucidation of molecular mechanisms [94], diagnosis and therapeutic procedures [95].

The central nervous system (CNS) is highly enriched in n-3 LC-PUFAs, of which DHA is the most important, participating in many crucial processes within the mammalian cells, as well as being the most abundant component of the neuron membrane, having important structural and functional roles. Membrane status affects the transfer of neuronal information, speed of signal transduction, and interaction with proteins, indicating the importance of this FA in order to gain appropriate functioning [96]. DHA is also the principal n-3 FA in cortical gray matter, representing ~15% of total FAs in the adult human prefrontal cortex (PFC). Moreover, other FAs like EPA, and less importantly DPA, are also present in the CNS, constituting around 1% of total brain FA composition [97] (and references within). Therefore, the control of the amount of DHA in our body, and especially in our liver and brain is really crucial for an optimal brain nutrition and functionality.

Alzheimer’s disease (AD) is a neurodegenerative disorder that produces severe cognitive impairment as it progresses. This pathology is the main neurological cause of dementia suffered by 36 million people worldwide, elderly adults in most cases (World Alzheimer Report 2011) [98]. The molecular phenotype is characterized by the accumulation of amyloid β peptide (Aβ) and aggregates composed of the hyperphosphorylated microtubule-associated tau protein [99].

Regarding lipids, DHA exerts a neuroprotective mode of action together with its lipoxygenase metabolite, Neuroprotectin D1. They have been shown to participate in many different processes including reductions in proinflammatory signalling (prostaglandin E2 synthesis), activation of important genes like *Bcl*-*2*, *Bcl*-*xl*, *Bfl*-*1*, which participate in antiapoptotic mechanism, and also reducing the expression of proapoptotic genes (*Bax, Bad*) [100]. In this sense, the relationship between DHA deficiency and neuropathology in the pre frontal cortex, and how n-3 FAs deficiency may increase vulnerability to patients suffering affective disorder have been reviewed [97]. For example, it has been observed that patients with major depressive disorder (MDD), or bipolar disorder (BD) exhibit a deficit of DHA in erythrocytes (MDD, −20%; BD −32%) relative to healthy controls [101].

Regarding AD, statistical differences in the amount of DHA in the liver of control subjects (324.83 ± 122.89) and those of patients with Alzheimer’s disease (204.64 ± 74.62) have been reported. These data correlated with both a lower amount of DHA in the brain, and a lower expression level of two genes involved in DHA biosynthesis in liver, *fds2* and *helo1* [102]. In post-mortem neocortex tissue, the profile of different lipids was investigated through GC–MS analysis. The results showed significant higher levels of FAs in AD neocortex region brain samples compared to non-AD neocortex region brain samples. A total of 9 FAs were elevated, with a marked increase in the percentage of DHA being observed in the cerebral region of AD patients [103]. This contrasted with most other reports of brain samples from AD individuals, which indicated a drop in DHA or an unaltered level in different regions of the brain [104, 105]. This increase may be due to the anti-inflammatory capacity of DHA, which may appear increased to activate the anti-inflammatory defence mechanism in AD brain.

FA profiles in plasma also seem to be important and were quantified and compared with three different cortical regions in brains of deceased participants of the Memory and Aging Project. The percentage of DHA was lower in both the mid-frontal cortex and superior temporal cortex of AD brains compared to non-impaired brains, but only in the phosphatidylserine fraction. Furthermore, in plasma the differences in FA profiles between AD and non-impaired samples was related to the free FA (lower oleic acid isomers and omega-6 FAs in AD) and phospholipids (lower omega-3 FAs in AD), suggesting that the level of DHA in plasma is directly related with AD [104]. On the other hand EPA, which commonly displays 250–300 times lower level in the brain compared to DHA, appears preferably esterified to phosphatidylinositol (PI) [106], and plays also an important role in AD. It was shown that higher plasma levels of EPA, but not DHA, were connected with lower gray matter atrophy, suggesting that EPA might play an important role in cognitive decline. Higher EPA levels were also found in the right amygdale, and may be associated with depressive symptoms [107].

Within the brain, there exist glial cells comprising microglia and astrocytes. Although the role of glial cells in AD pathology is not clear, they are supposed to give support to neurons by removing amyloid β peptides from the brain, and also modulate inflammation [108]. Dysfunction of these cells may be related to AD development through accumulation of amyloid β peptide. Since DHA can modulate glial cell activity, it was considered that dietary supplement of DHA may positively modulate the effects of Alzheimer [109]. It has been reported that the polymerization of Aβ monomers incubated with DHA was significantly inhibited (40%). DHA was also able to inhibit fibril formation [110]. These authors also reported that the level of DHA in the hippocampus of the Aβ-infused AD rats was significantly lower than in control, and how the subsequent DHA administration markedly helped to significantly increase the level of DHA. A mechanism of fighting AD is the clearance of amyloid β, which has been suggested to delay or even prevent AD symptoms. A very interesting recent investigation showed that DHA and EPA mediate degradation of Aβ by stimulating and boosting the gene expression of insulin-degrading enzyme (*ide*), resulting in a higher amount of IDE, the major Aβ-degrading enzyme secreted into the extracellular space of neuronal and microglial cells [111].

Another interesting fact regarding Aβ and its plasmatic accumulation has been recently described. The offspring of rats exposed to n-3 and n-6 PUFA enriched diets showed a significant increase in plasmatic Aβ levels compared to control when their diet was n-6 PUFA-enriched, and decreased levels when a high n-3 PUFA diet was supplied. This accumulation is related with hyper-activation of the HPA axis, alteration of cortical monoamine content, and an induced depressive- and anxiety-like state in adult offspring. Altogether these data endorse the positive and protective role of an n-3 rich diet [112]. The relationship between soluble Aβ and depression in AD is still considered as a hotspot in AD investigation; in fact, the effects of soluble Aβ accumulation and depression have been widely reported highlighting the strong relationship [113–115].

Hashimoto and collaborators have investigated the effect of DHA in human neuroblastoma cells (SH-SY5Y). These cells display a considerably lower content of DHA (2.15 ± 0.25 mol%) compared to that found in normal neuronal cells (DHA > 10% of total FAs). The authors observed that DHA inhibits the in vitro fibrillation of Aβ_25−35_ with a parallel inhibition of neurotoxicity induced by the formation of fibrils. In the presence of DHA, the degree of fibrillation significantly decreased by about 43%. However, when the Aβ_25−35_-treated SH-SY5Y cells were examined after treatment with DHA, the data indicated that the addition of DHA to the cells prevented the toxicity of Aβ_25−35_ [116].

Furthermore, it has been reported that *fat*-*1* transgenic mice, which express more n-3 PUFAs (total n-3 PUFAs in WT: 17.51, in *fat*-*1* mice: 23.45), are more resistant to intra cerebroventricular injection of Aβ, which induces neuronal cell loss and impairment of hippocampus-dependent memory [117]. Similar to the results found in *fat*-*1* mice, the clearance of interstitial Aβ was much faster in those mice treated with fish oil, compared to untreated mice. Such a protective effect has been suggested to occur through an activation of the AQP4-mediated glymphatic system to promote interstitial Aβ clearance [118]. DHA, as a fundamental component of neuronal membranes, has been widely studied as an active compound to treat Alzheimer effects [119]. It has been shown that the novel hydroxyl-derivative of DHA (OHDHA) has a strong therapeutic potential to treat AD. It has been demonstrated that administration of OHDHA increases the concentration of PUFAs-containing phospholipids (e.g. DHA or EPA-containing phospholipids). The increase in n-3 PUFAs in transgenic mouse model of AD was accompanied by a parallel reduction in amyloid-β accumulation, which is strongly correlated with synaptic degeneration, and with memory failure and learning deficiencies [98] (and references within). DHA asserts its protective role through its stereoselective bioactive product Neuroprotectin D1 (NPD1), which exerts anti-inflammatory and survival signalling in Alzheimer’s disease [120].

Parkinson’s disease (PD), was first medically described as a neurological syndrome by James Parkinson in 1817. It is a progressive neurodegenerative disease characterized by loss of dopaminergic neurons containing neuromelanin in the substantia nigra pars compacta (SNpc) [121]. The common symptoms include tremor, rigidity, bradykinesia and postural insecurity, with dementia and depression observed in the advanced stages of the disease. It is known that a decline in FA concentration is observed in neurodegenerative diseases, such as PD, and it could be expected that n-3 PUFAs may exert neuroprotective action in PD, as previously shown in Alzheimer’s disease. It has been described that PUFAs are known to play an important role in lowering anxiety and also improving cognitive functions in non-human primates [122].

One study investigated the effect of the neurotoxin 1-methyl-4-phenyl-1,2,3,6-tetrahydropyridine (MPTP) when given to mice (from 2 to 12 months of age) fed a diet high in n-3 PUFAs. It was observed that the rich n-3 PUFA diet protected against the MPTP-induced decrease in dopamine, and its metabolite dihydroxyphenylacetic acid in the striatum. These data suggest that a high n-3 PUFAs dietary intake might exert neuroprotective actions in an animal model of Parkinson disease [123].

Within LC-PUFAs, DHA is the most important one for the brain; recently, in an experimental mouse model of PD the effects of the neurotoxin MPTP administrated together with DHA were evaluated. The results indicated that DHA has protective effects on dopaminergic neurons in this MPTP-induced experimental model [124]. Transgenic animal models have also been used to test the protective effect of high levels of n-3 PUFAs. Bousquet and collaborators published that the expression of the *fat*-*1* transgene (which encodes the enzyme n-3 PUFA desaturase that catalyzes the n-6 to n-3 PUFA conversion) in mice increased the cortical n-3:n-6 PUFA ratio (+28%) compared to control. This modification is less effective than a dietary supplementation, since a 10-month exposure to an n-3 PUFA-enriched diet increased the n-3:n-6 PUFA ratio by as much as 92%. Regarding neuroprotection in animal models of PD using MPTP, the data suggest that dietary intake of a preformed DHA supplement is more effective than the expression of the *fat*-*1* transgene [125].

The preventing effect of DHA on dopaminergic lesion was also studied by determining the levels of tyrosine hydroxylase (TH) in MPTP and MPTP + DHA treated cells [126]. There was a 65% decline in the number of TH-immunopositive neurons in the substancia nigra one week after MPTP administration and the number of TH-positive neurons significantly increased in the MPTP + DHA group compared to the MPTP control group. DHA may block the conversion of MPTP to MPP+ or prevents the uptake of MPP+ into dopaminergic terminals. This study suggested that dietary supplementation with DHA may be a potential means for delaying the onset of PD and/or the rate of progression [126].

One-third of PD patients suffer depression that may lead to worse health outcomes, and a decreased quality of life. Anxiety, apathy and anhedonia further complicate PD outcomes. DHA was tested as a therapy to reduce these symptoms. It was observed that 75% of PD patients treated with DHA and EPA (DHA, 800 mg/day, EPA 290 mg/day) for 6 months showed a reduction of at least 50% on the Hamilton Rating Scale for Depression, compared with only 25% into the placebo group. These data suggest that a combination of DHA and EPA can reduce the depressive symptoms of PD [127]. A similar study regarding depression using the Hoehn and Yahr scale did not however show a significant variation in all groups during a 3 month trial using fish oil supplements (containing omega-3 FAs). However, when the patients were evaluated for depression symptoms using the Montgomery–Asberg Scale, it was observed that after 3-month supplementation, fish oil groups, taking or not antidepressant medication, exhibited a reduction in depression symptoms when compared to mineral oil groups [128].

It has been demonstrated that DHA stimulates the production of syntaxin 3 (STX3), a plasma membrane protein that has an important role in the growth of neuritis in PC12 cells and hippocampal neurons cultures. This protein is directly activated by ARA, but DHA also serves as an activator of STX3. Therefore, DHA can efficiently substitute dietary ARA in activating syntaxin 3 [129]. Regarding dietary omega-3 FAs, it has been shown that the combination of voluntary exercise potentiated the effects of DHA after 12-day dietary supplementation with a resulting increase of STX3. Levels of STX3 were stimulated separately by both DHA diet (150% compared to control) and exercise (126% compared to control). It should be highlighted that the combination of DHA diet and exercise regimens showed an up-regulation of STX3 levels (186% of control) [130]. These authors have also published that DHA and exercise positively affect the levels of the NMDA receptor subunit NR2B, which is important for synaptic function underlying learning and memory. Together DHA and exercise boost NR2B levels up to 169% compared to control, while DHA and exercise separately were able to elevate the levels of NR2B to 145 and 123% respectively, compared to the controls [130]. All these data suggest that exercise can influence the effect of DHA on neuron membrane function, and help to maintain synaptic and cognitive function by supporting membrane stability.

Amyotrophic lateral sclerosis (ALS) is a rare group of neurological diseases that mainly involve the nerve cells (neurons) responsible for controlling voluntary muscle movement. The disease is progressive, in which symptoms get worse year after year. Unfortunately, there is neither a cure nor an effective treatment to halt, or reverse, the progression of the disease. Membranes from motor neurons and other neurons in the CNS possess the highest amounts of PUFAs. They play an important role in the maintenance and function of the membrane by acting through a variety of molecular mechanisms [131]. A recent study in a mouse model of ALS (G93A-mutated human superoxide dismutase) has identified a relationship between neuronal loss and DHA levels. In this mouse model a significant decrease in DHA-containing phosphatidylcholines (PCs), in the terminal stage of ALS was shown [132]. Using MALDI-IMS analysis, the authors observed that the mean signal intensities corresponding to PC (diacyl-16:0/22:6) in the anterior horn in L5 spinal cord sections of 22-week-old mice model were significantly lower than those in the age-matched control, and this lost was related with motor neuron cell death.

It is known that ALS related protein oxidative damage affects FA concentrations specifically the n-3 series [133]. For example DHA levels were 30% lower in spinal cord samples from ALS patients compared to control. This data surprisingly contrasts with the significant increase (18%) observed in frontal cortex samples. TDP-43 protein, a transcriptional repressor, is found as cytosolic aggregates in sporadic ALS. It is known that in ALS, TDP-43 protein pathologies are related with oxidative stress in which ERK1/2 pathway is involved [134]. It has been published that cytosolic aggregates of TDP-43 protein associated with ALS produced in the spinal cord showed a lower expression of some DHA synthesizing enzymes, such as ACAA1, FADS1 and COX1. The authors analyzed the tissular concentration of these enzymes observing that in the spinal cord, the enzymes involved in FA β-oxidation like ACAA1 and ACAA2, as well as those related to early steps of FA desaturation, like FADS2, were all increased in the analyzed ALS samples (ranging between 20 to 110%) [135].

A dietary intake of omega-3 is really important in ALS, as well as in Alzheimer or Parkinson. In a large analysis of a total of 995 ALS patients fed with an omega-3 PUFA enriched diet, this intake was associated with a risk reduction of 34% for ALS. Further analysis of the individual omega-3 PUFAs established that intakes of ALA and marine n-3 PUFAs, were each associated with a lower ALS risk [136].

Inflammatory processes that destroy motor neurons in the spinal cord, brain stem and cortex during ALS could be reduced by DHA administration due to its potent anti-inflammatory properties. It is known that DHA increases total glutathione levels in microglia cells, thus enhancing their antioxidative capabilities. In parallel, DHA causes a reduction of pro-inflammatory cytokines, such as IL-6 or TNF-α. Furthermore, Poly I:C, a toll-like receptor (TLR) agonist, has the capacity of increasing the secretion of IL-6 produced by microglia. Poly I:C is able to mimic a viral or bacterial infection through activation of TLR pathway, which are considered the first line of defence against viral and bacterial pathogens. Stimulation of an immortalized mouse brain cell line (EOC20 microglia) with Poly I:C causes an increase in IL-6; however, a 24 h pre-treatment with DHA resulted in a lowering of IL-6 to undetectable levels, indicating the inhibiting capacity of DHA of pro-inflammatory cytokines [137].

In another mouse model however, n-3 PUFAs could also act negatively, stimulating the symptoms of ALS progression as well as lipid peroxidation compared to control [138]. Feeding pre-symptomatic mice (G39A-SOD1) EPA (300 mg/kg/day), for 6 weeks increased lipid peroxidation. Higher amounts of 4-hydroxy-2-hexenal, a toxic and reactive aldehydic intermediate formed by nonenzymatic peroxidation of n-3 PUFAs, were detected in the entire gray matter of sporadic ALS [139]. The increment of 4-hydroxy-2-hexenal could result in an increased oxidative stress in microglia and could also be related with a decrease in microglia cell number [138].

Altogether this information indicates that n-3 PUFAs, particularly DHA, are crucial for the development, maintenance and function of the CNS and should be included in our diet. Moreover, further investigation should be carried out in order to determine the LC-PUFAs molecular and physiological mechanisms participating in neurodegenerative diseases affecting humans.

## Diet as a tool to acquire functional lipids and to maintain health

Food is part of our daily activity, and of vital importance for the maintenance of life. The relevance of omega-3 LC-PUFAs, particularly EPA and DHA, in human nutrition and health has been emphasized. Briefly, LC-PUFAs including ARA are the predominant components of the 75–88% of the phospholipids constituting the cell membrane bilayer in animals. They play a central role in almost all physiological and biochemical events within the cells, and therefore, in maintaining optimal cellular function [140, 141].

It is widely accepted that for attaining a healthy status a balanced omega-6 to omega-3 ratio in our diet is more essential than the absolute amounts of individual FAs. In the diet of early humans, the proportion between both groups of FAs was maintained close to 1:1. However, in present-day Western diets, characterised by a high intake of saturated fats, n-6 FAs, detrimental trans-FAs, and elevated consumption of sugars, this ratio has risen to 20:1 or even higher, leading to continuous daily intakes that are far away from the optimal acquisition of healthy n-3 FAs. This imbalance favours obesity and has been proven to be prothrombotic and proinflammatory [25, 142], which stimulates a myriad of inflammatory dependent ailments, such as arthritis, cancer, hypertension, diabetes, asthma, atherosclerosis, Alzheimer, Parkinson, allergies and many more [27, 28, 143].

Although mammals, including humans, possess the biochemical machinery and enzymes to fabricate LC-PUFAs from their C18 precursors, they are unable to de novo biosynthesize the essential fatty acids ALA and LA, which can only be attained through diet. Although some ALA is converted to the longer-chain omega-3 FAs, the extent of this conversion is modest. Thus, in vivo studies in humans reported that ca. 5% of ALA was converted to EPA and ca. 0.5% of ALA was converted to DHA [20], indicating the low ability to produce these important FAs. Interestingly, another recent report shows that although the conversion of ALA into DHA is not very efficient (<1%), the addition of supplemental ALA, which did not significantly increase the detected amounts of DHA in plasma or erythrocytes unlike EPA that did augment upon ALA feeding, appeared to be sufficient to sustain brain function. Moreover, it was determined that extremely low ALA dietary intakes are required to significantly affect brain DHA levels and function, assuming however, that the neurological impairments observed with ALA deficiency are caused by the decrease of DHA in brain [144]. Overall, it is widely accepted that the biosynthetic pathway in humans does not provide sufficient levels of ALA because of being, together with LA, essential FAs, to meet EPA and DHA demands and therefore, a dietary intake of preformed omega-3 LC-PUFAs is needed.

Recommendations of consumption and/or supplementation of these omega-3 FAs are specific to particular groups of age and physiological status, and still require further study to be adequately established. However, most health bodies and Government agencies recommend consuming between 250 and 1000 mg of EPA + DHA per day for adults, to maintain health and well-being [145]. Particularly, the Food and Agriculture Organization (FAO) of the United Nations jointly with the World Health Organization (WHO), and the European Food Safety Authority (EFSA) recommend a daily EPA + DHA intake of at least 250 mg. On the other hand, the lower limit for the health of a general adult population set by the UK Government is 450 mg EPA + DHA/day [47, 146, 147]. These amounts should be increased up to 1–1.5 g/day for cardiovascular disease (CVD) risk reduction or patients with hypertriglyceridemia [145]; although the real benefit of this recommendation is sometimes controversial [148].

Human milk is a source of LA, ALA, ARA, EPA, DHA and other LC-PUFAs. While the level of ARA remains relatively constant, that of DHA is more variable and depends on maternal diet and lifestyle. During pregnancy, increased maternal dietary requirements of omega-3 FAs occur because of fetal accretion [149]. Enhanced maternal n-3 FA intakes during pregnancy and lactation may result in an increase in the duration of gestation and infant size at birth, reducing the incidence of preeclampsia and postpartum depression. In addition, some evidences from both observational studies, and interventional trials suggest that a higher n-3 LC-PUFAs supply was associated with beneficial effects on infant growth and neurodevelopment [150]. Pregnant and lactating women achieving an average daily dietary DHA intake of at least 150–200 mg up to about 1200 mg, prolonged gestation, increased birth weight, and reduced the risk of preterm delivery before 34 weeks of gestation by 31% in all pregnancies or by 61% in high-risk pregnancies. No adverse effects were registered apart from some cases of belching or unpleasant taste, even at the highest dietary levels assayed of 1.2 g/day of DHA or 2.7 g/day of total n-3 LC-PUFAs [150]. In a recent review, the relationship between reduced maternal consumption of n-3 PUFAs, and the prevalence of childhood and adolescence neuropsychiatric condition including autistic spectrum disorders (ASD), attention deficit disorders (ADH), schizophrenia spectrum disorders (SSD), and depression and anxiety disorders has been highlighted [151].

In summary, a minimum daily intake of 300 mg/day of EPA + DHA, of which 200 mg/day are DHA or up to 700–1000 mg of EPA + DHA/day is highly recommended for a more favourable pregnancy and lactation [145].

The diets of vegetarian (those avoiding all meat) and vegan (those completely avoiding animal-based products) have been associated with markedly lower levels of DHA in plasma phospholipids. In vegetarians, ALA supplementation was demonstrated to increase the proportion of EPA but not DHA. It would thus appear that only supplementation with preformed DHA reliably increases tissue DHA. On the other hand, although vegans had zero intake of preformed DHA, it was nevertheless present in blood phospholipids, suggesting basal conversions from ALA to DHA that may be up-regulated by the absence of—and down-regulated by the presence of—dietary preformed DHA [19]. In line with this, breast milk DHA cannot be increased with the addition of ALA or other DHA precursors to the diet, hence the breast milk of strict vegans contains the lowest DHA amount compared to that of fish-eaters or vegetarians [152].

Fish oil is the largest source of EPA and DHA, while eating fish constitute the major way of ingesting these functional FAs in human diets. A modest consumption of 2–3 servings of fish per week, particularly oily fish such as salmon, tuna, herring, mackerel or sardines, can be enough to meet most intake recommendations for healthy status (Table 1). There has been a continuous increase in the apparent fish consumption (kg per capita) from 9.9 kg in the 1960s to 14.4 in the 1990s, and to beyond 20.0 kg in 2015 [153]. This increase correlates with the concomitantly higher omega-3 FAs intake within this period. This increment is in part due to the significant growth in fisheries and aquaculture products as a whole, and to the raising awareness of the world’s population to the benefits of fish consumption for human health. However, fish is a declining resource, with a disquieting 58.1% of all fish stocks being overfished, and more than 26% of stocks fished at biologically unsustainable levels [153]. With capture fishery production being relatively static at around 80–90 million tonnes per year since the late 1980s, aquaculture has been proposed by the FAO as the potential complement to meet the growing demand for marine based alimentation. In fact, the aquaculture contribution to the supply of fish for human consumption overtook that of wild-caught fish in 2014 for the first time [153]. At present, one of the major challenges for the aquaculture industry is the environmental sustainability of the activity. New sustainable and environmentally friendly ingredients for aquafeeds are needed, that do not compromise the nutritional benefits of fish consumption for humans.

**Table 1:** Amounts of total fat, EPA, DHA and EPA + DHA (g/100 g of serving) in fish species of different origin and processing

Fish name	Total fat	EPA	DHA	EPA + DHA
Atlantic mackerel (cooked)	17.8	0.504	0.699	1.203
Greenland halibut (cooked)	17.7	0.670	0.504	1.178
Chinook salmon (cooked)	13.4	1.010	0.727	1.737
Atlantic salmon (farmed)	12.4	0.690	1.457	2.147
Atlantic herring (cooked)	11.6	0.909	1.105	2.014
Red salmon (cooked)	11.0	0.530	0.700	1.230
Pacific sardine (canned in tomato)	10.5	0.532	0.865	1.397
European anchovy (canned in vegetable oil)	9.7	0.763	1.292	2.055
Atlantic salmon (wild, cooked)	8.1	0.411	1.429	1.840
Rainbow trout (farmed and cooked)	7.2	0.334	0.820	1.154
Red tuna (fresh or cooked)	6.3	0.363	1.141	1.504
Rainbow trout (wild and cooked)	5.8	0.468	0.520	0.988
Sword fish (cooked)	5.1	0.138	0.681	0.819
Atlantic and Pacific halibut (cooked)	2.9	0.091	0.374	0.465
Channel catfish (wild, cooked)	2.9	0.100	0.137	0.237
European pollock (cooked)	1.9	0.076	0.162	0.238
Mere fish various sp. (cooked)	1.3	0.035	0.213	0.248
Shrimp various sp. (cooked)	1.1	0.171	0.144	0.315
Atlantic cod (cooked)	0.9	0.004	0.154	0.158
Tuna (canned in brine)	0.8	0.047	0.223	0.270

Fish recommendation must also be balanced with concerns about environmental pollutants. Larger fish predators may contain significant levels of methyl mercury, and other potentially harmful contaminants, such as polychlorinated biphenyls (PCBs) and dioxins that bioconcentrate in the aquatic food chain. Children and pregnant or lactating women may be at a higher risk of mercury intoxication from fish consumption and thus, it seems prudent to avoid consuming large quantities of higher fish predators. The consumption of a variety of fish species is the best approach to both minimizing mercury exposure and increasing omega-3 FAs intake [48].

None fish-eaters, or those who just want an extra boost, may ingest omega-3 supplements. Marine microorganisms are the major source for consumption and for the production of encapsulated fish oil. Fish oil is commercially available mainly as soft gel capsules often including added vitamins, antioxidants, and various flavours. However, capsules should be taken under medical supervision, because taking fish oil can cause a number of gastrointestinal disturbances, including belching, heartburn, nausea and loose stools. In addition, people under the age of 18 years, and pregnant or lactating women should not take fish oil capsules unless under the supervision of a doctor. To minimize possible adverse side effects, these supplements should be stored in the refrigerator or freezer and kept away from light, and should be taken when they are cold in order to reduce undesirable stomach upset symptoms.

As fish oil production is currently declining, other sources of omega-3 LC-PUFAs are currently being evaluated. Micro and macroalgae, some of them with valuable nutritional FA profiles, are already under industrial exploitation after optimization of culture and growth medium conditions [154]. Other promising sources of omega-3 essential FAs such as zooplankton, commonly known as “krill”, fungi, and genetically modified plants must overcome many challenges, such as cost, environmental concerns, consumer acceptance, viability and sensory quality prior to their consolidation [148].

## Conclusions

Undoubtedly lipidomics has allowed tremendous advances in understanding and determining the true importance of lipids and FAs in many physiological and molecular mechanisms implicated in the establishment of healthy or diseased status.

There is compelling evidence that omega-3 LC-PUFAs, particularly EPA and DHA, and an adequate balance of omega-6/omega-3 FAs play a determinant role in most physiological and biochemical processes occurring in cells and organisms, having great significance in decreasing the risk of many diseases or even resolving their inherent inflammation condition. This has been broadly driven by the impressive development of technologies applied to lipidomics, and the subsequent understanding of the underlying molecular mechanisms of lipid metabolism.

Nutritional recommendations, based on epidemiological evidence, have been advocated by many health authorities, and agencies to reduce both, the excessive intake of omega-6 PUFAs, and the very high omega-6/omega-3 ratio of current diets. Thus, consumption of higher amounts of fish, the main source of biologically important omega-3 LC-PUFAs in combination with natural antioxidants is strongly recommended. In this sense, and although a more fine-tuning is still needed to particular groups of age and physiological status, 450–500 mg of EPA + DHA per day is suggested for general health condition of the adult population. This could be met with a weekly ingestion of 2–3 portions of oily fish. Increased amounts of up to 1 to 3–4 g/day of omega-3 LC-PUFAs should be ingested during pregnancy and lactation or to prevent most cardiovascular, neurodegenerative and proinflammatory disorders.
